# Polysulfide Immobilization and Sulfur Conversion Kinetics Promotion via a Tetrathiafulvalene–Crown Ether COF@Graphene Layer for High‐Rate Lithium–Sulfur Batteries

**DOI:** 10.1002/smll.74240

**Published:** 2026-06-16

**Authors:** Kai Sun, Tsukasa Irie, Samim Reza, Kohki Sasaki, Mika Nozaki, Tokuhisa Kawawaki, Yujun Fu, Dequan Liu, Ranjit Thapa, Saikat Das, Deyan He, Yuichi Negishi

**Affiliations:** ^1^ School of Materials and Energy Lanzhou University Lanzhou Gansu China; ^2^ Institute of Multidisciplinary Research for Advanced Materials Tohoku University Sendai Japan; ^3^ Department of Physics SRM University AP Amaravati Andhra Pradesh India; ^4^ Centre for Computational and Integrative Sciences SRM University – AP Amaravati Andhra Pradesh India

**Keywords:** covalent organic framework, cycling stability, electrocatalytic effect, lithium polysulfides, lithium‒sulfur batteries, shuttle effect, sulfur redox reaction

## Abstract

The rational design of conversion‐kinetics‐promoting framework architectures offers a powerful strategy to overcome the polysulfide shuttling and sluggish conversion kinetics that limit lithium–sulfur (Li–S) batteries. Here, we report a new covalent organic framework, TUS‐44, constructed from tetrathiafulvalene‐based 4,4',4'',4'''‐([2,2'‐bi(1,3‐dithiolylidene)]‐4,4',5,5'‐tetrayl)tetraaniline and benzo[18]crown‐6‐derived 4,4′,4′′,4′′′‐(6,7,9,10,17,18,20,21‐octahydrodibenzo[*b*,*k*][1,4,7,10,13,16]hexaoxacyclooctadecine‐2,3,13,14‐tetrayl) tetrabenzaldehyde linkers. The resulting imine‐linked, π‐conjugated framework integrates heteroatom‐rich coordination sites within an electron‐delocalized π‐conjugated backbone, establishing a hierarchical, site‐specific interaction network (N > O > S) that governs Li^+^ coordination, polysulfide anchoring, and dynamic charge redistribution. When coupled with conductive graphene to form an interfacial TUS‐44@G architecture, the hybrid layer functions as a chemisorptive, conversion‐kinetics‐promoting interface that regulates soluble polysulfides, facilitates interfacial charge‐transfer, and stabilizes intermediate species throughout cycling. This synergistic chemical–electronic coupling yields remarkable electrochemical performance: a high reversible capacity of 1455.7 mA h g^−1^ at 0.2 A g^−1^, excellent rate capability of 773 mA h g^−1^ at 10 A g^−1^, and outstanding durability with 0.034% capacity fading per cycle over 1000 cycles at 5 A g^−1^. A Li–S pouch cell incorporating TUS‐44@G further delivers an outstanding initial energy density of ∼674 Wh kg^−1^ at 0.05 A g^−1^ (sulfur loading 44.558 mg), underscoring the practical potential of architecting conversion‐catalytic framework interfaces for high‐energy, durable Li–S batteries.

## Graphical abstract

A polysulfide‐regulating covalent organic framework (TUS‐44) integrating tetrathiafulvalene and crown‐ether linkers forms an electron‐delocalized, ion‐coordinative network that synergistically mediates Li–S redox chemistry. When interfaced with graphene, the TUS‐44@G layer functions as a catalytic and chemisorptive interface, enabling efficient polysulfide regulation, fast charge‐transfer, and durable high‐energy lithium–sulfur battery performance.

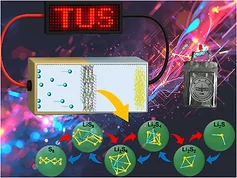

## 1 Introduction

As of 2025, the global energy landscape is undergoing a transformative shift, driven by the escalating demand for sustainable and high‐performance energy storage solutions. Lithium‐ion batteries (LIBs) presently lead the energy storage sector; however, Benchmark Mineral Intelligence data cited in the U.S. DOE's National Blueprint for Lithium Batteries 2021–2030 indicate that U.S. production will reach only ∼148 GWh by 2028—covering less than half of the projected demand [1]. Their widespread adoption stems from their high energy density, long cycle life, and technological maturity, enabling reliable large‐scale manufacturing. However, as the demand for electric vehicles (EVs) and renewable energy storage systems intensifies, the limitations of LIBs become more apparent. These include relatively low theoretical capacity and the reliance on scarce and expensive materials, such as cobalt and nickel, which pose sustainability and supply chain challenges. In response to these limitations, lithium–sulfur (Li–S) batteries [2, 3, 4, 5, 6, 7, 8, 9, 10, 11, 12] have emerged as a promising alternative, offering a theoretical specific capacity of 1672 mA h g^−1^ for sulfur and a significantly higher energy density compared to conventional LIBs [5]. Moreover, sulfur is abundant, low‐cost, and environmentally benign, making Li–S batteries an attractive option for large‐scale energy storage applications. Despite these advantages, the practical deployment of Li–S batteries is severely constrained by the notorious polysulfide shuttle effect—a complex parasitic phenomenon in which soluble high‐order lithium polysulfides (LiPSs; Li_2_S_4_–Li_2_S_8_), generated during the discharge process, readily dissolve into the electrolyte and diffuse between the cathode and the lithium anode [13, 14, 15, 16, 17]. This uncontrolled migration leads to several detrimental consequences: continuous loss of active sulfur species and severe capacity fading; parasitic redox reactions at the lithium surface that consume both electrolyte and active lithium; formation of unstable solid–electrolyte interphase (SEI) layers; and pronounced self‐discharge and coulombic inefficiency. Moreover, the repeated dissolution–redeposition cycles induce morphological degradation of the cathode, exacerbate polarization, and accelerate corrosion and passivation of the lithium metal anode—collectively undermining the long‐term electrochemical stability and practical viability of Li–S systems. To address these intertwined challenges, extensive efforts have focused on rationally designing advanced cathode architectures, optimized electrolytes, and functional separator modifications to suppress LiPS diffusion and accelerate redox kinetics. At the cathode level, diverse sulfur–host composites have been explored to physically confine and chemically anchor soluble polysulfides while maintaining efficient charge transport. Porous carbon materials such as mesoporous carbons, graphene frameworks, and carbon nanotube networks provide high surface area and conductivity, offering mechanical accommodation for volume expansion and capillary trapping of LiPS species [18, 19]. However, their intrinsically nonpolar surfaces rely mainly on weak van der Waals interactions, which are insufficient to fully suppress shuttle under practical sulfur loadings. To enhance chemical affinity, polar inorganic hosts—such as TiO_2_, MnO_2_, Co_3_O_4_, MoS_2_, and transition‐metal nitrides—have been incorporated, leveraging Lewis acid–base interactions to immobilize LiPS and catalyze their conversion [20, 21, 22, 23, 24]. Despite their improved trapping capability, the limited electronic conductivity and high density of these materials often undermine the gravimetric energy density and rate performance. Conducting polymers such as polypyrrole, polyaniline, and PEDOT have also been employed as electrochemically functional matrices that combine mechanical flexibility with chemical anchoring sites [25, 26, 27]. Yet, their electrochemical and structural stability deteriorate over extended cycling, particularly in ether‐based electrolytes. More recently, reticular materials including metal–organic frameworks (MOFs) and covalent organic frameworks (COFs) have emerged as promising sulfur hosts, owing to their tunable porosity, rich coordination sites, and structural regularity [28, 29]. Nevertheless, many pristine frameworks can face challenges such as limited structural stability under repeated lithiation–delithiation, pore blockage at high sulfur loadings, or restricted electrolyte accessibility, which may compromise long‐term performance if not carefully engineered. Electrolyte engineering offers a complementary strategy to mitigate polysulfide dissolution and protect the lithium anode. The incorporation of LiNO_3_ as a passivating additive remains a widely adopted approach, forming a stable inorganic–organic SEI that suppresses parasitic reactions and enhances coulombic efficiency [30]. However, LiNO_3_ is gradually consumed during cycling, leading to declining protection and shortened lifespan. Alternative additives—such as LiFSI, functional organics, and redox mediators—have been proposed to stabilize interfaces and regulate LiPS solvation structures [31, 32], while high‐concentration or localized high‐concentration electrolytes (HCE/LHCE) further modulate polysulfide chemistry through solvent–ion coordination [33, 34]. Yet, these formulations often incur higher viscosity, cost, and limited ion mobility, rendering them less practical for large‐format cells. In parallel, separator and interlayer modifications have emerged as efficient design tools for restricting LiPS migration without fundamentally altering cell architecture. Carbonaceous interlayers (graphene, CNTs, carbon nanofibers) enhance electron transport and reutilize trapped LiPS [35, 36], while polar ceramic coatings (e.g., TiO_2_, Al_2_O_3_, MnO_2_) chemically adsorb anionic intermediates [37, 38]. Polymer‐functionalized membranes and polysulfide‐regulating MOF or COF coatings have also demonstrated promise by integrating ionic selectivity and catalytic conversion sites at the separator interface [39, 40, 41]. However, these strategies frequently introduce additional interfacial impedance or inactive mass, reducing overall energy density. Moreover, the delicate balance between strong LiPS binding and reversible redox regeneration remains difficult to achieve—excessive adsorption can immobilize active species, whereas weak interactions permit diffusion losses. Consequently, despite remarkable progress, the development of a unified molecular‐level strategy that integrates high conductivity, selective chemisorption, and catalytic redox mediation within a structurally robust platform remains a central challenge in Li–S chemistry.

COFs [42, 43, 44, 45, 46, 47, 48, 49, 50, 51, 52, 53, 54, 55] have attracted intense interest as a platform for addressing the central mechanistic problems of Li–S chemistry because they combine permanent, atomically tunable porosity with molecular precision in functionality and topology. COFs are crystalline, covalently linked polymers whose pores, functional groups and backbone redox properties can be designed synthetically, making them well suited to play multiple, complementary roles in Li–S cells — as sulfur hosts that confine active material and buffer volume change, as interfacial coatings or interlayers that chemically immobilize dissolved polysulfides while promoting charge transport, and as selective, ion‐conducting membranes that discriminate against polysulfide crossover without hindering Li^+^ flux [56, 57]. Early experimental demonstrations established the feasibility of using ordered organic frameworks to host sulfur and improve retention of active species: impregnation of elemental sulfur into nanoporous COFs was shown to produce sulfur electrodes with improved capacity retention relative to unconfined sulfur, illustrating the benefit of well‐defined pore environments for trapping soluble intermediates [58]. Building on this concept, a large and rapidly growing body of work has explored how specific COF chemistries and architectures affect polysulfide binding energy, Li‐ion accessibility, and electronic coupling between the host and electroactive sulfur species. In particular, electrochemically functional COFs — frameworks that incorporate electrochemically addressable units (carbonyls, quinones, imine/azomethine redox centers, tetrathiafulvalene motifs, etc.) into the backbone — are attractive because they add a catalytic/electronic dimension to simple physical confinement. Rather than acting as passive traps, such frameworks can transiently accept or donate electrons during the multi‐electron sulfur conversion sequence, lowering kinetic barriers for polysulfide conversion and facilitating more uniform Li_2_S nucleation and re‐oxidation. This principle has been demonstrated across several recent reports in which carbonyl‐rich or otherwise redox‐functionalized COFs show enhanced reversible capacity, improved rate behavior, and suppressed shuttle vs. non‐functionalized hosts [59, 60, 61]. Beyond acting as cathode hosts, COFs have been exploited as separator coatings and membrane layers that combine size‐exclusion, electrostatic selectivity, and catalytic functionality. Precisely functionalized COF nanosheets or ion‐tuned COF membranes have been reported to repel anionic polysulfides (via tailored pore size and tethered anionic/cationic groups), while maintaining or even enhancing Li^+^ conduction; such ion‐channel or anion‐tethered COF membranes reduce self‐discharge and improve capacity retention under practical loadings [62, 63, 64]. These device‐level demonstrations underscore that COFs can be engineered as active, conversion‐kinetics‐mediating interfaces rather than passive barriers. Notwithstanding these advances, important practical challenges remain before redox‐active COFs can be widely adopted in commercial‐scale Li–S cells. First, the intrinsic electronic transport across extended COF lattices is still limited by the insulating nature of most organic linkages, leading to sluggish redox kinetics and uneven current distribution at high areal loadings. Achieving delocalized π‐conjugation or built‐in charge‐transport pathways through judicious linker and node design thus remains a central challenge. Second, framework chemical stability in aggressive Li–S electrolytes and under repeated lithiation/delithiation must be rigorously confirmed, as certain linkages (e.g., imine, boronate ester) may hydrolyze or undergo side reactions during prolonged cycling. Third, pore accessibility at high sulfur fill fractions and under lean‐electrolyte conditions can be compromised by pore blockage or by the formation of insulating Li_2_S deposits. Finally, scalable processing of thin, uniform COF films or coatings compatible with existing electrode‐manufacturing workflows remains an engineering hurdle. These limitations point to clear directions for future work: (i) design intrinsically conductive or metalated redox COFs that obviate heavy conductive additives, (ii) engineer robust linkages and sterically accessible binding motifs that survive long‐term cycling, and (iii) develop scalable deposition and hybridization strategies (e.g., COF@graphene, COF–polymer blends, COF nanosheet inks) that deliver the sought‐after balance of conductivity, catalytic function, and low inactive mass. Addressing these gaps will be essential to translate the considerable promise of electrochemically functional COFs into durable, high‐energy Li–S devices.

**SCHEME 1:**
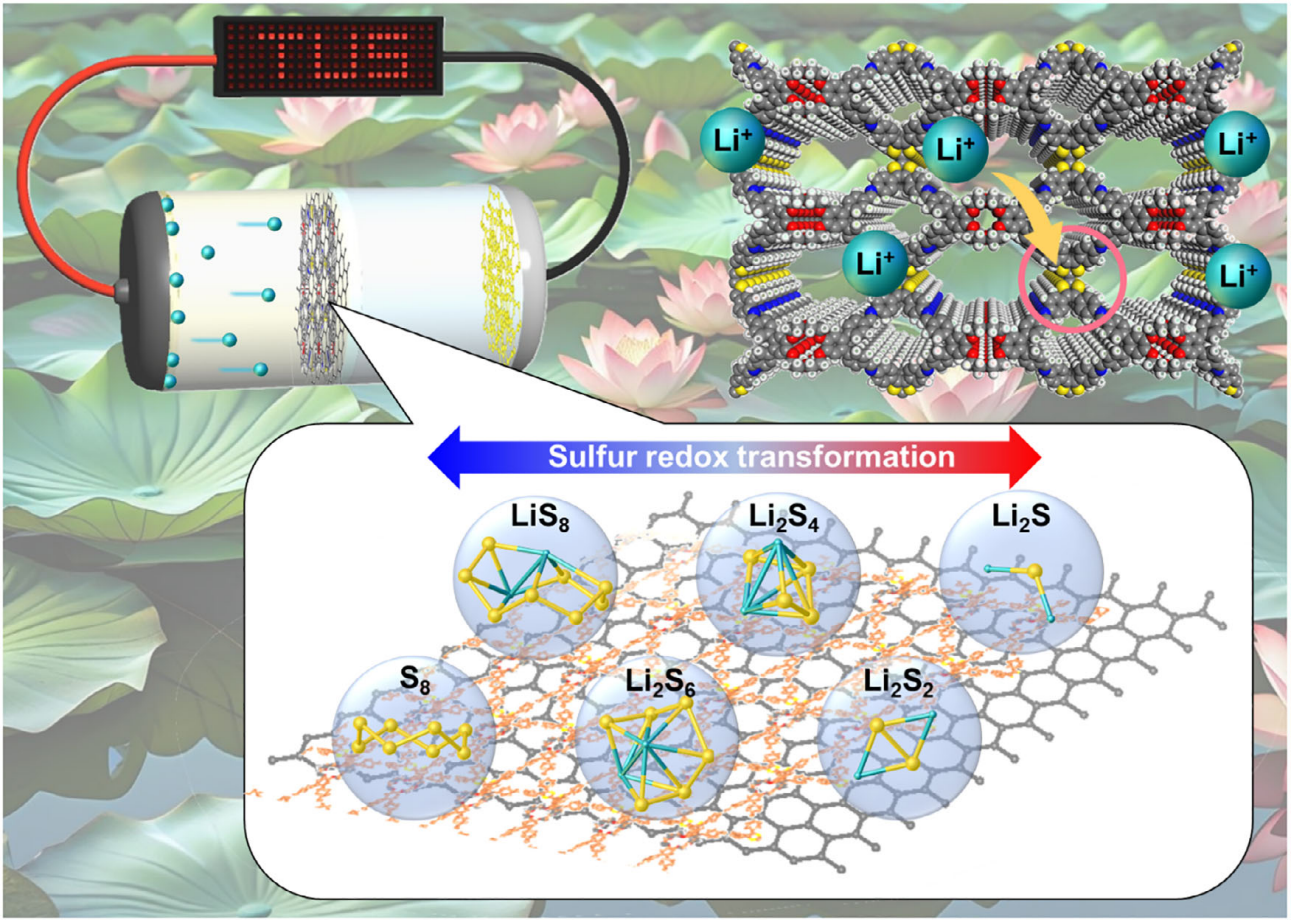
Working concept of the Li–S battery integrated with a TUS‐44@G functional layer that mediates site‐specific adsorption and catalytic conversion of sulfur species, promoting reversible transformation between S_8_, LiPSs, and Li_2_S_2_/Li_2_S with minimized polysulfide migration, accelerated redox kinetics, and enhanced cycling stability.

In this context, we report a new polysulfide‐regulating COF, TUS‐44, rationally constructed to integrate electronic conductivity, polysulfide regulation, and interfacial conversion‐kinetics mediation within a unified framework–conductor hybrid. The COF architecture combines a tetrathiafulvalene (TTF)‐based polysulfide‐regulating tetraaniline node with a benzo[18]crown‐6‐derived tetrabenzaldehyde linker, forming an imine‐linked, π‐conjugated network that is both electron‐delocalized and structurally adaptable. The framework encodes a hierarchical interaction environment in which imine nitrogen sites serve as primary Li^+^ anchoring centers, crown‐ether oxygen atoms facilitate auxiliary Li^+^ coordination and transport, and sulfur‐rich TTF units contribute to electronic coupling and redox mediation. When integrated with conductive graphene to form a TUS‐44@G interfacial layer, the resulting hybrid layer achieves synergistic chemical–electronic coupling, simultaneously enabling conversion catalysis, polysulfide confinement, and rapid charge equilibration across the interface (Scheme 1). This cooperative mechanism delivers exceptional electrochemical performance, including a high reversible capacity of 1455.7 mA h g^−1^ at 0.2 A g^−1^, superior rate capability of 773 mA h g^−1^ at 10 A g^−1^, and outstanding long‐term stability with only 0.034% capacity fading per cycle over 1000 cycles. A Li–S pouch cell further exhibits a high initial energy density of ∼674 Wh kg^−1^ at 0.05 A g^−1^, highlighting the practical promise of conversion‐catalytic framework interfaces for high‐energy, durable Li–S batteries.

## 2 Results and Discussion

### 2.1 Crystallographic, Porosity, and Stability Analyses of the Bex‐Topology Framework TUS‐44

**FIGURE 1:**
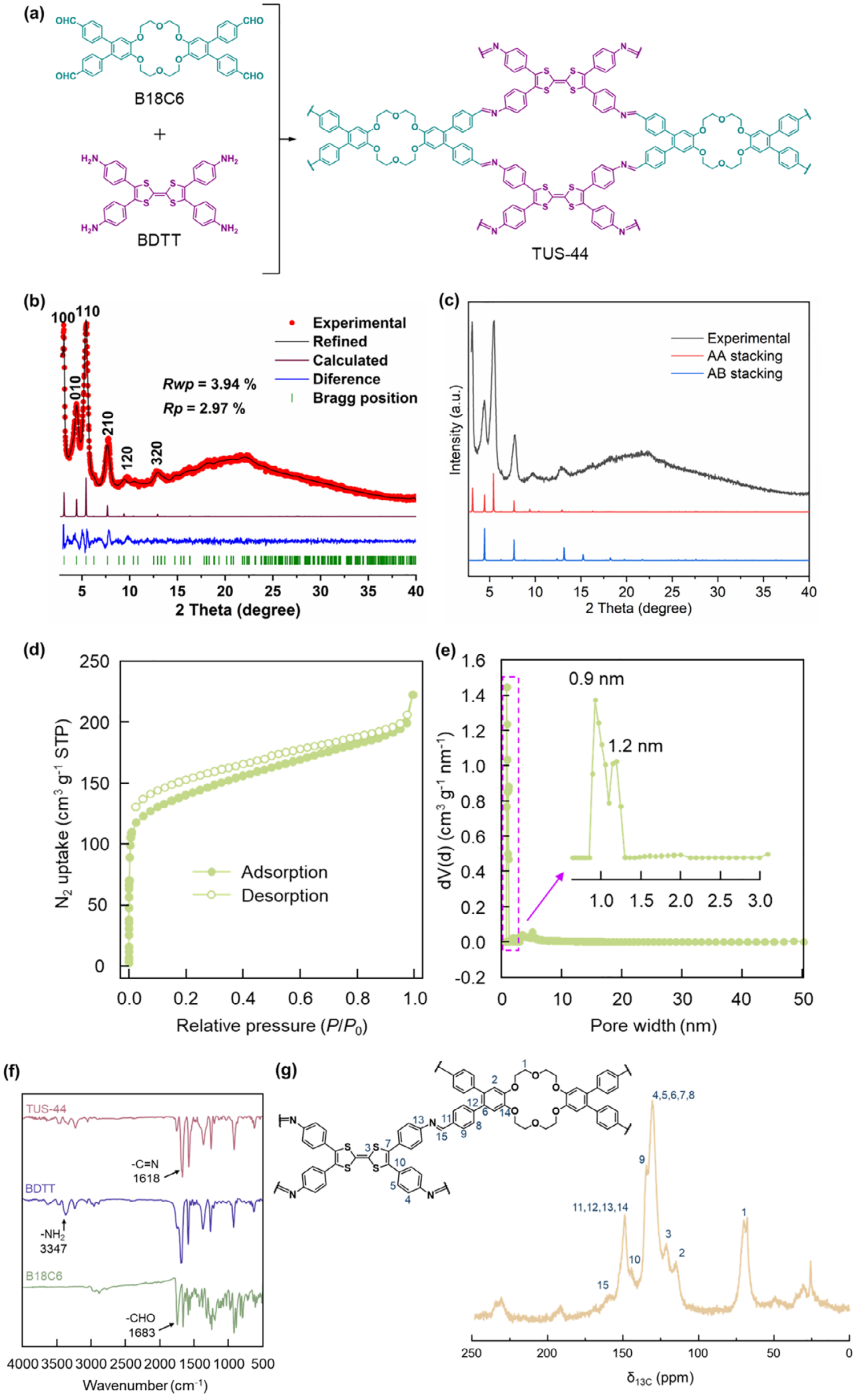
Design and structural features of TUS‐44: (a) Controlled assembly of B18C6 and BDTT linkers affording the sulfur‐rich, π‐conjugated framework TUS‐44, (b) Experimental XRD pattern (red circles), Pawley refinement (black curve), calculated pattern (purple curve), difference plot (blue curve) between the experimental and refined patterns, and Bragg positions (green ticks). (c) Comparison of experimental PXRD with simulated profiles for AA and AB‐stacked models, (d) N_2_ sorption isotherms at 77 K and (e) corresponding pore‐size distribution from QSDFT, for TUS‐44. (f) FT‐IR spectra of TUS‐44, BDTT, and B18C6. (g) Solid‐state ^13^C CP/MAS NMR spectrum of TUS‐44.

TUS‐44 was synthesized via a solvothermal Schiff‐base condensation between the tetrabenzaldehyde macrocycle 4,4′,4′′,4′′′‐(6,7,9,10,17,18,20,21‐octahydrodibenzo[*b*,*k*][1,4,7,10,13,16]hexaoxacyclooctadecine‐2,3,13,14‐tetrayl) tetrabenzaldehyde (B18C6) and the tetraaniline building block 4,4',4'',4'''‐([2,2'‐bi(1,3‐dithiolylidene)]‐4,4',5,5'‐tetrayl)tetraaniline (BDTT) in an equimolar ratio. Following systematic optimization of solvent and catalyst conditions, the use of *o*‐dichlorobenzene (*o*‐DCB) with 6 m aqueous acetic acid as a catalyst at 120°C for 72 h afforded TUS‐44 as a brown, highly crystalline solid in 62% isolated yield (Figure 1a). The choice of a large, rigid tetrabenzaldehyde and an electron‐rich tetrathiafulvalene‐derived tetraaniline (BDTT) directs formation of extended imine bonds and enforces a two‐dimensional (2D) reticular lattice with well‐defined microporous channels. The long‐range order and topology of TUS‐44 were established by comparison of experimental powder x‐ray diffraction (PXRD) patterns with simulated diffractograms from energy‐minimized structural models. Geometry optimization was performed using the Forcite module (*Materials Studio* 7.0) [65] and classical force‐field methods to generate candidate stacking modes and 2D nets. Pawley refinement of the experimental PXRD against the AA‐stacked bex net model produced excellent agreement (*R*_p_ = 2.97%, *R*_wp_ = 3.94%), confirming that TUS‐44 crystallizes in an orthorhombic lattice (space group *P*MM2 (No. 25)) with unit‐cell parameters *a* = 28.2832 Å, *b* = 19.9426 Å, *c* = 3.6946 Å, and angles α = β = γ = 90° (Table S1). Distinct low‐angle Bragg reflections at 2θ = 3.08°, 4.42°, 5.50°, 7.78°, 9.84°, and 12.88° were indexed to the (100), (010), (110), (210), (120), and (320) planes, respectively (Figure 1b). The close match between experimental and simulated profiles, together with a minimal difference curve, demonstrates the long‐range periodicity and high structural fidelity of the proposed AA‐stacked bex model (Figure 1b,c); alternative AB stacking scenarios were simulated (Figure S10), but their XRD patterns did not reproduce the measured diffraction (Figure S9), supporting the AA registry as the correct packing motif. Mechanistically, the bex net in TUS‐44 is constructed according to the *D*_2h_ + *D*_2h_ topological principle, wherein the BDTT linker serves as a four‐directional node, and the B18C6 tetrabenzaldehyde provides the complementary four‐armed connector, together generating a 2D reticulation with well‐defined, periodic pores. The AA stacking aligns π‐conjugated linkages from adjacent layers, producing an interlayer spacing of ≈3.69 Å (*c*‐axis) that favors through‐plane *π–π* interactions and facilitates electron delocalization perpendicular to the sheet plane.

Porosity was characterized by N_2_ adsorption–desorption at 77 K after thermal activation at 120°C for 8 h. The isotherm exhibits a steep uptake at very low relative pressures (P/P_0_< 0.1), diagnostic of intrinsic microporosity (Figure 1d). Multipoint Brunauer–Emmett–Teller (BET) analysis in the low‐pressure region yields a specific surface area of 516 m^2^ g^−1^ (Figure S2). Quenched solid density functional theory (QSDFT) pore‐size analysis using a slit/cylindrical hybrid model shows a narrow, well‐defined pore distribution with dominant peaks centered at ≈0.9 and 1.2 nm (Figure 1e), in excellent agreement with the pore apertures of ≈0.87 and 1.16 nm computed from the structural model (Figure S1). The close correspondence between model‐derived and experimentally measured pore sizes confirms that the designed 2D bex topology is faithfully realized in the framework, providing uniform microporous channels that facilitate site‐specific adsorption and strong chemical anchoring of LiPSs.

Formation of the imine (C═N) linked framework was confirmed by complementary spectroscopies. Fourier‐transform infrared (FT‐IR) spectroscopy of TUS‐44 shows the emergence of a strong C═N stretching band at 1618 cm^−1^ along with pronounced attenuation of the BDTT N–H stretching band (originally at 3347 cm^−1^) and the B18C6 aldehyde C═O band (originally at 1683 cm^−1^), consistent with near‐quantitative consumption of starting functional groups (Figure 1f). Solid‐state ^13^C cross‐polarization magic angle spinning (CP/MAS) NMR spectroscopy exhibits a distinctive resonance at ≈159 ppm assignable to the imine carbon, further corroborating successful Schiff‐base condensation and covalent network formation (Figure 1g). Elemental analysis (observed C: 72.97; H: 4.33; N: 4.54; O: 7.78; S: 10.39) is in close accord with theoretical stoichiometry (C: 72.28; H: 4.22; N: 4.22; O: 9.64; S: 9.64), with minor deviations plausibly attributable to residual solvent inclusion or experimental uncertainty in oxygen quantification—overall supporting the expected framework composition.

**FIGURE 2:**
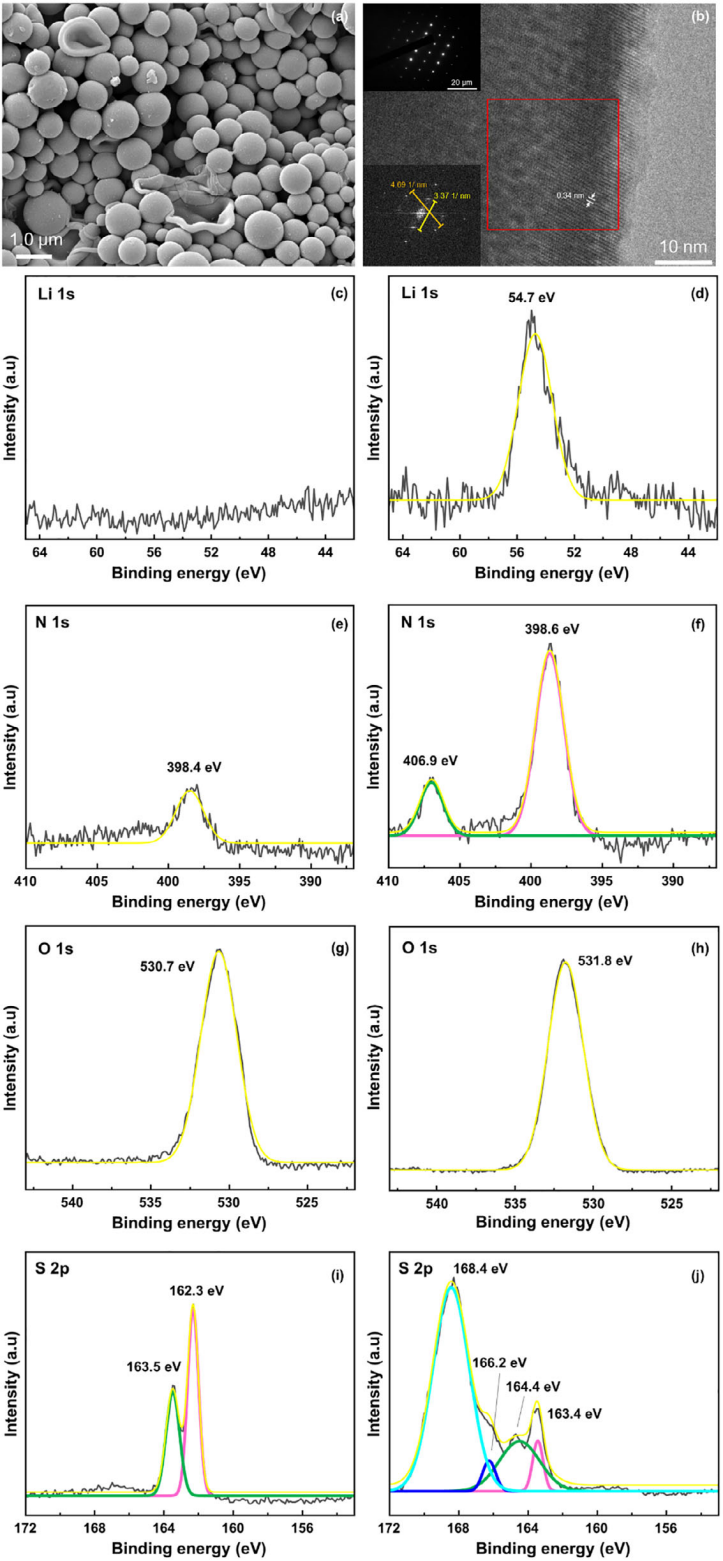
Morphological, structural, and surface chemical characterization of TUS‐44 and its Li–S redox interfaces: (a) SEM and (b) HRTEM images of TUS‐44. The upper inset in (b) shows the SAED pattern, while the lower inset presents the FFT pattern obtained from the red‐marked region. High‐resolution XPS spectra for (c,d) Li 1s, (e,f) N 1s, (g,h) O 1s, (i,j) S 2p collected before and after cycling, respectively, with the TUS‐44@G modified separator.

Scanning and transmission electron microscopy (SEM/TEM) reveal uniform, well‐defined spherical crystallites of TUS‐44 with narrow size dispersity (Figure 2a and Figures S3 and S4). High‐resolution TEM (HRTEM) shows lattice fringes with a measured *d*‐spacing of ≈0.34 nm, assignable to the (001) interlayer plane, and fast Fourier transform (FFT)/selected area electron diffraction (SAED) patterns with discrete spots that confirm long‐range crystalline order (Figure 2b). These microscopy data corroborate the PXRD‐derived picture of layered, periodically stacked 2D sheets with coherent interlayer registry. Thermogravimetric analysis (TGA) under N_2_ demonstrates that TUS‐44 is thermally robust, showing negligible mass loss up to ≈360°C (Figure S7). Chemical stability tests—immersion in the Li–S battery electrolyte solvents 1,3‐dioxolane (DOL) and 1,2‐dimethoxyethane (DME) for 7 days—showed no detectable loss of crystalline order by PXRD (Figure S8), indicating excellent resistance to solvent swelling and bond hydrolysis under the tested conditions. The high thermal decomposition onset and solvent resilience make TUS‐44 well suited for interfacial roles in Li–S cells where long‐term exposure to organic electrolytes and elevated local temperatures can otherwise degrade host materials.

The combination of a bex topology, AA registry, narrow and uniform micropores, and aligned π‐conjugation imparts three key functional attributes that are relevant to Li–S electrochemistry: (*i*) site‐specific adsorption—uniform channel size and exposed tetrathiafulvalene motifs provide electronic and steric complementarity to confine and chemically stabilize polysulfide species; (*ii*) enhanced interfacial conductivity—appreciable *π–π* stacking and electronic coupling between layers lower interfacial charge‐transfer resistance and support catalytic redox activity; and (*iii*) robustness under operational conditions—thermal and solvent stability ensure structural integrity during repeated redox cycling and electrolyte exposure. These structural attributes rationalize the strong adsorption, catalytic mediation, and cycling benefits observed experimentally when TUS‐44 is employed as an interfacial host in Li–S cells.

### 2.2 Morphological Characterization, Surface Chemistry Evolution, and Polysulfide Permeation Resistance

To construct an interfacial layer capable of both electronic conduction and conversion‐kinetics mediation, composite TUS‐44@G/PP membranes were fabricated through a facile vacuum filtration process, in which a homogeneous dispersion of TUS‐44 and graphene in *N*‐methyl‐2‐pyrrolidone (NMP) was filtered onto a Celgard polypropylene (PP) separator and subsequently dried to yield a uniform, flexible composite film. This procedure produced a conformal coating of the TTF‐functionalized COF on the PP support, forming an integrated architecture that functions as a robust barrier and catalytic interface for Li–S batteries. Morphological investigation by SEM revealed distinct microstructural differences between the pristine and modified membranes (Figure S6). The bare PP separator (Figure S6a) displays an open, fibrous network with abundant micropores, typical of Celgard‐type polyolefin membranes, which facilitates ion conduction but lacks any functional surface sites to suppress polysulfide diffusion. In sharp contrast, the surface of the TUS‐44@G/PP composite (Figure S6e) is completely covered by a continuous, compact, and uniform TUS‐44@G layer, effectively sealing the intrinsic pores of PP. The cross‐sectional SEM image (Figure S6f) reveals a bilayered configuration with comparable thicknesses for the TUS‐44@G coating and the underlying PP substrate, indicating intimate interfacial contact. The analogous G/PP membrane (Figure S6d) exhibits a similar morphology but without the molecular‐level redox functionality of the COF, underscoring the dual role of the TUS‐44@G layer as both a physical and chemical barrier.

To elucidate the chemical origin and site‐specific nature of polysulfide immobilization within the TUS‐44@G interface, x‐ray photoelectron spectroscopy (XPS) was performed on the separator before and after 50 electrochemical cycles (Figure 2c–j). The analysis reveals a coordinated evolution of Li, N, O, and S electronic environments, providing direct spectroscopic evidence for the dominant interaction pathways. The N 1s spectrum of pristine TUS‐44@G/PP (Figure 2e) exhibits a single peak at 398.4 eV, characteristic of imine (C═N) nitrogen within the conjugated COF backbone, confirming the integrity of the Schiff‐base linkage. After cycling (Figure 2f), the N 1s region evolves into two distinct components at 398.6 and 406.9 eV. The peak at ∼398.6 eV corresponds to residual, uncoordinated imine nitrogen, whereas the high‐binding‐energy component at 406.9 eV reflects strongly electron‐deficient nitrogen species. This pronounced positive shift is indicative of direct coordination between Li^+^ and imine nitrogen (N–Li interaction), accompanied by significant charge withdrawal from nitrogen. Importantly, this spectroscopic signature is fully consistent with DFT results (Section 2.5), which reveal that N sites exhibit the strongest and most balanced adsorption toward Li_2_S*_n_* species, with substantial charge‐transfer from Li to the COF framework. The emergence of the high‐binding‐energy N component therefore provides direct experimental validation that imine nitrogen serves as the primary anchoring site for lithium polysulfides, rather than a passive structural unit. The Li 1s spectrum further corroborates this interaction. No Li signal is observed in the pristine state (Figure 2c), while after cycling (Figure 2d), a distinct peak appears at 54.7 eV, characteristic of Li in Li–S/Li–N coordinated environments. Notably, the presence of this peak, together with the N 1s shift, indicates that Li^+^ ions are not solely associated with sulfur species but are partially stabilized through coordination with nitrogen sites, forming interfacial Li–N–S configurations. This multi‐site interaction promotes strong immobilization while preserving redox reversibility. The O 1s spectra show only a minor shift from 530.7 to 531.8 eV after cycling (Figure 2g,h), suggesting relatively weak electronic perturbation of crown ether oxygen atoms. This observation aligns well with DFT results, which indicate that O sites preferentially interact with short‐chain polysulfides but exhibit weaker affinity toward long‐chain Li_2_S*_n_* species. Therefore, oxygen plays a secondary, auxiliary role, primarily contributing to Li^+^ solvation and ion transport rather than dominant chemisorption. The S 2p spectra provide further insight into both framework stability and polysulfide chemistry. In the pristine material (Figure 2i), the peaks at 162.3 and 163.5 eV correspond to the spin–orbit doublet (S 2p_3/2_ and S 2p_1/2_) of neutral C–S–C sulfur within the TTF units, confirming the intact organosulfur framework. After cycling (Figure 2j), the S 2p envelope becomes significantly broadened and resolves into multiple components. Peaks in the 162–164 eV range are assigned to reduced sulfur species (Li_2_S/Li_2_S*_n_*), while new features at 166–168 eV correspond to oxidized sulfur species such as thiosulfate/polythionate formed during interfacial redox reactions. Crucially, although TTF sulfur sites participate in Li–S interactions, the absence of a pronounced binding‐energy shift comparable to N 1s indicates that S atoms primarily contribute to electronic coupling and redox mediation rather than acting as the dominant Li^+^ anchoring centers. These spectroscopic signatures, in direct agreement with DFT analysis, establish a clear interaction hierarchy within TUS‐44, wherein imine nitrogen sites act as primary chemisorption centers (N > O > S), oxygen sites contribute to auxiliary Li^+^ coordination and transport, and sulfur sites predominantly facilitate electronic mediation and redox coupling.

To further substantiate the polysulfide‐blocking capability of the composite separator, visual diffusion experiments were performed in H‐type permeation cells (Figure S11). When Li_2_S_6_ solution was placed in the left chamber and blank electrolyte in the right, the pristine PP membrane allowed rapid polysulfide diffusion, evident from the right‐side electrolyte turning yellow within 1 h and darkening over 6 h. Conversely, the TUS‐44@G/PP membrane exhibited exceptional impermeability, with the right chamber remaining colorless even after 48 h. This pronounced suppression of LiPS migration is attributed to the combined physical confinement and chemical adsorption imparted by the TTF‐functionalized COF layer, wherein nitrogen‐centered coordination sites dominate Li^+^ anchoring and polysulfide immobilization, while sulfur‐rich TTF units contribute primarily to electronic coupling through π–S–assisted charge‐transfer, and oxygen sites assist Li^+^ transport within the interlayer, while graphene ensures a continuous conductive pathway for electron transport. These results reveal that the TUS‐44@G interlayer functions as a polysulfide‐regulating chemisorptive barrier that dynamically regulates polysulfide transport and transformation, stabilizes reactive intermediates, and promotes reversible redox kinetics—collectively accounting for its superior electrochemical performance in Li–S batteries.

### 2.3 Electrochemical Performance and Reaction Kinetics of Li–S Cells Enabled by TUS‐44@G Interlayer Architecture

**FIGURE 3:**
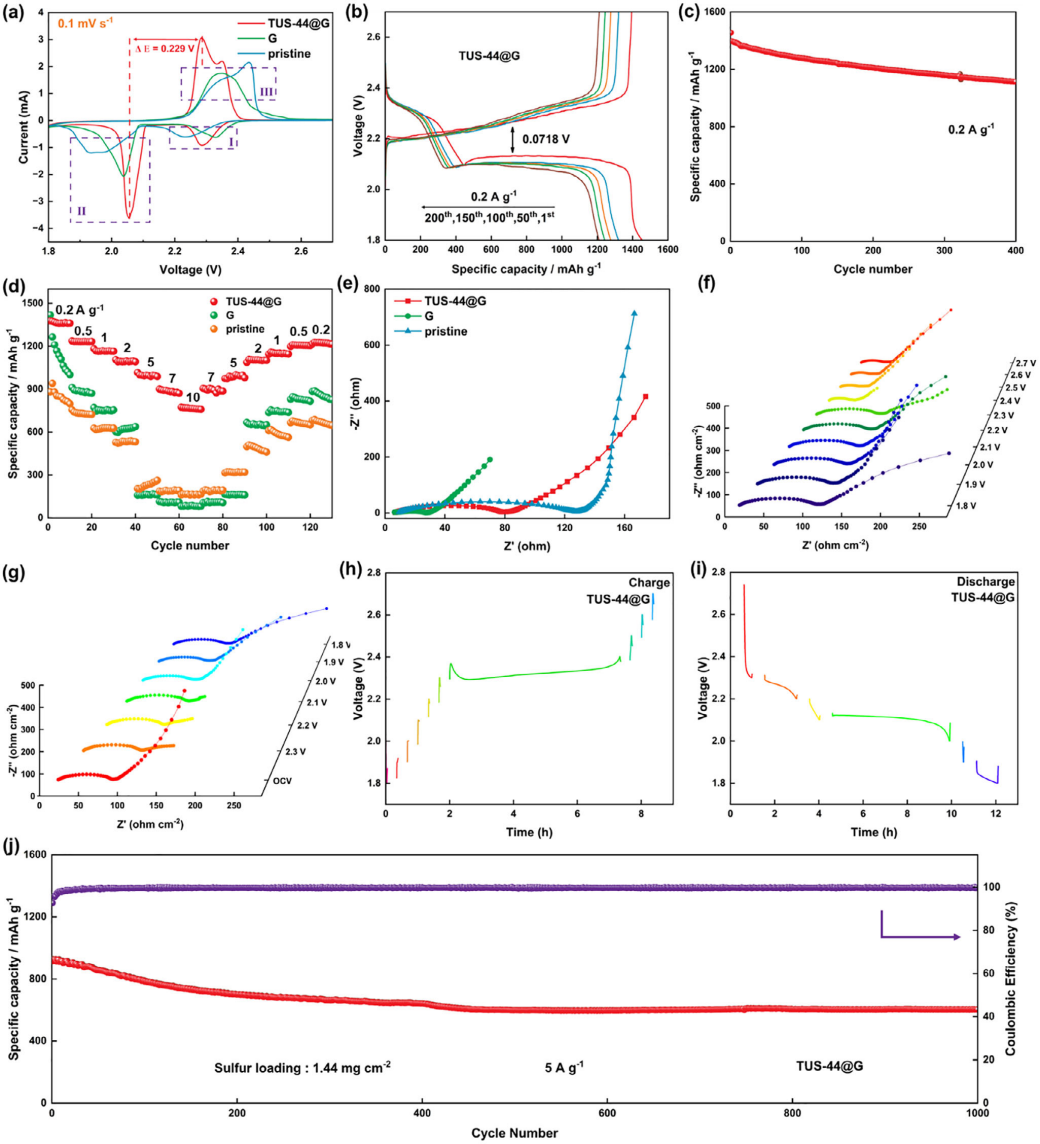
Electrochemical behavior and interfacial dynamics of TUS‐44@G‐regulated Li–S chemistry: (a) Cyclic voltammograms reflecting reversible sulfur–polysulfide transitions, (b) galvanostatic charge/discharge profiles at 0.2 A g^−1^ across multiple cycles, (c) cycling stability over 400 cycles at 0.2 A g^−1^, (d) rate performance at various current densities, and (e) EIS spectra comparing interfacial resistances in cells with different interlayers. Potential‐dependent EIS spectra recorded during (f) charge and (g) discharge track interfacial evolution. Galvanostatic (h) charge and (i) discharge curves of the TUS‐44@G‐based cell display distinct potential plateaus associated with stepwise S_8_ ↔ LiPSs ↔ Li_2_S_2_/Li_2_S transformations. (j) Long‐term cycling and coulombic efficiency at 5 A g^−1^ sustained over 1000 cycles.

The electrochemical characteristics of Li–S batteries incorporating the TUS‐44@G‐modified interlayer were evaluated using CR2032‐type half‐cells. Cyclic voltammetry (CV) was first conducted at a scan rate of 0.1 mV s^−1^ in the voltage range of 1.8–2.7 V vs. Li/Li^+^ to probe the redox reversibility and assess the kinetics of sulfur conversion reactions (Figure 3a). CV analysis is critical in Li–S systems as it provides direct insight into the stepwise redox transformations between sulfur (S_8_), soluble lithium polysulfides (Li_2_S*_n_*, 4 ≤ *n* ≤ 8), and insoluble Li_2_S_2_/Li_2_S species, thereby revealing the catalytic efficacy and charge‐transfer behavior of the functional interlayer. The TUS‐44@G‐based cell exhibits two well‐resolved cathodic peaks at 2.28 and 2.05 V, corresponding respectively to the reduction of S_8_ to soluble long‐chain polysulfides and the subsequent conversion to insoluble Li_2_S_2_/Li_2_S. In the anodic scan, two distinct oxidation peaks at 2.28 and 2.34 V are observed, which are assignable to the re‐oxidation of Li_2_S/Li_2_S_2_ into soluble polysulfides and the final regeneration of elemental sulfur, respectively. The sharpness and high current intensity of these redox peaks, coupled with the remarkably low polarization potential of only 0.229 V, underscore the outstanding electrochemical reversibility and accelerated electron‐transfer kinetics enabled by the polysulfide‐regulating TUS‐44 framework. The extremely low polarization implies minimal energy loss during the sulfur redox transformations, reflecting rapid Li^+^ diffusion and efficient catalytic mediation at the TUS‐44@G interface. This indicates that the imine‐linked, heteroatom‐cooperative TUS‐44 domains—dominated by nitrogen‐centered Li^+^ anchoring sites—not only serve as strong adsorption centers for LiPS intermediates but also facilitate their bidirectional redox conversion through redox‐coupled charge transport.

The galvanostatic charge–discharge profiles at 0.2 A g^−1^ (Figure 3b) display two distinct discharge plateaus at 2.3 and 2.1 V, corresponding to the successive reduction of S_8_ to Li_2_S*_n_* and further to Li_2_S_2_/Li_2_S, in good agreement with the CV results. The charge plateau centered at ∼2.2 V reflects the oxidation of Li_2_S/Li_2_S_2_ back to S_8_. The exceptionally low overpotential (ΔV = 71.8 mV) between charge and discharge plateaus reveals highly reversible sulfur redox chemistry and low kinetic barriers for LiPS conversion. Importantly, the voltage profiles maintain their shape and plateau integrity even after 200 cycles, indicating that the TUS‐44@G interlayer effectively suppresses polarization buildup, mitigates the shuttle effect, and sustains a uniform electrochemical environment. At a lower rate of 0.05 A g^−1^, the cell exhibited a comparably low polarization voltage of 95.8 mV (Figure S23), reinforcing the conclusion that the TTF‐functionalized framework promotes facile charge‐transfer and fast Li^+^ diffusion. The cycling stability of the TUS‐44@G‐integrated cell is particularly noteworthy, delivering an initial discharge capacity of 1455.7 mA h g^−1^ and maintaining 1113.5 mA h g^−1^ after 400 cycles (retention = 76.5%) (Figure 3c). Such durable capacity retention can be attributed to the heteroatom‐cooperative framework of TUS‐44, in which imine nitrogen sites act as primary coordination centers for Li^+^ anchoring, supported by auxiliary oxygen coordination for Li^+^ transport and sulfur‐mediated electronic coupling for redox kinetics. These Li–S interactions anchor the polysulfide intermediates at the conductive framework interface, effectively suppressing their diffusion and ensuring efficient redox conversion during cycling. The TTF units simultaneously participate in rapid electron mediation, facilitating reversible polysulfide transformations and uniform Li_2_S deposition. Meanwhile, the benzo[18]crown‐6 linkers enhance the overall framework flexibility and electrolyte accessibility, facilitating homogeneous ion transport and mitigating concentration gradients across the interlayer. The rate performance profiles (Figure 3d) further highlight the kinetic superiority of the TUS‐44@G‐based system. The cell delivered high reversible capacities of 1377.7, 1237.2, 1182.2, 1106.5, 1015.9, 900.2, and 773 mA h g^−1^ at current densities of 0.2, 0.5, 1, 2, 5, 7, and 10 A g^−1^, respectively. When the current rate was stepped back to 0.2 A g^−1^, the capacity almost fully recovered (1217.1 mA h g^−1^), corresponding to 88.3% retention, confirming excellent structural robustness and rapid LiPS conversion kinetics. Even at an ultra‐high rate of 10 A g^−1^, the achieved capacity of 773 mA h g^−1^ surpasses the values of graphene‐only (∼81.2 mA h g^−1^) and Celgard (∼161 mA h g^−1^) systems, demonstrating the pronounced catalytic efficiency of the TUS‐44@G interface in enabling fast polysulfide redox reactions.

Electrochemical impedance spectroscopy (EIS) was employed to probe the interfacial charge‐transfer behavior and reaction kinetics of the Li–S cells under various electrochemical states. The Nyquist plots of the cells assembled with different separators (Celgard, graphene, and TUS‐44@G) exhibit a depressed semicircle in the high‐to‐medium frequency region followed by a sloping line in the low‐frequency region, characteristic of the combined charge‐transfer and ion‐diffusion processes typical of Li–S batteries (Figure 3e). Among them, the TUS‐44@G‐modified cell displays a distinctly smaller semicircular diameter than the pristine PP separator, confirming a markedly reduced charge‐transfer resistance (*R*_ct_ = 44.24 Ω) compared to that of the PP‐based cell (*R*_ct_ = 91.56 Ω). This improvement stems from the synergistic combination of conductive pathways and conversion‐promoting sites provided by the TUS‐44 framework. Although the graphene‐based interlayer exhibits the lowest absolute *R*_ct_ (36.48 Ω) owing to its highly conductive carbon matrix, the TUS‐44@G configuration achieves an optimal balance between electronic conductivity, conversion‐kinetics mediation, and interfacial ion transport. This cooperative effect manifests as more stable and reversible electrochemical performance during prolonged cycling. The enhanced interfacial wettability of the TUS‐44@G layer further contributes to this improvement. Contact angle measurements demonstrate that the electrolyte contact angle of TUS‐44@G (24.3°) is markedly lower than that of Celgard (35.4°), indicating enhanced electrolyte affinity and improved ionic wetting across the interfacial region. Although slightly higher than that of pristine TUS‐44 (19.8°), the composite configuration ensures optimal synergy between the TTF‐functionalized framework and conductive graphene network, enabling uniform electrolyte infiltration and effective Li^+^ transport pathways. Such improved surface wettability not only facilitates intimate electrolyte contact but also minimizes interfacial impedance buildup during prolonged cycling.

To gain deeper insight into the dynamic evolution of the interfacial reactions, EIS measurements were further performed under different potentials during the charge–discharge process, ranging from 1.8 to 2.7 V (Figure 3f,g). Each spectrum consists of an incomplete capacitive arc in the high‐frequency region and a linear Warburg‐type diffusion tail at low frequencies, reflecting the coupled charge‐transfer and mass‐transport processes. A distinct voltage‐dependent modulation of the semicircle diameter is observed: at higher potentials (2.7–2.3 V), corresponding to the reduction of long‐chain Li_2_S*_n_*, the impedance remains relatively small, indicating facile redox kinetics and efficient electron transfer across the cathode–interlayer–electrolyte interface. As the discharge potential decreases to 2.1 V or below, the semicircle diameter markedly increases, signifying a sharp rise in *R*_ct_ associated with the nucleation and subsequent deposition of insoluble Li_2_S_2_/Li_2_S species. This stage represents the kinetically sluggish step of the sulfur reduction sequence and is the principal source of polarization in Li–S systems. The impedance evolution in the TUS‐44@G‐based cell faithfully mirrors this multistep redox mechanism, underscoring that the interlayer establishes a stable, conductive, and catalytically active microenvironment capable of modulating intermediate conversion processes. The overall impedance remains consistently lower than that of the pristine PP‐based cell throughout the potential range, confirming enhanced charge‐transfer kinetics and reduced interfacial barriers. The TTF units in TUS‐44 mediate reversible electron exchange with polysulfide species, while the benzo[18]crown‐6 linkers promote structural robustness and facilitate electrolyte infiltration. These cooperative effects result in uniform Li_2_S nucleation, efficient reoxidation during charging, and overall suppression of polarization—consistent with the minimal voltage hysteresis observed in the galvanostatic charge–discharge profiles (Figure 3b).

Figure 3h,i illustrate the representative galvanostatic charge and discharge profiles of the TUS‐44@G‐based Li–S cell, revealing distinct and well‐defined reaction plateaus characteristic of the multistep redox conversion between sulfur and LiPSs. During the charging process (Figure 3h), the voltage initially rises from ∼1.9 V and exhibits a pronounced peak near 2.35 V, corresponding to the oxidation of insoluble Li_2_S_2_/Li_2_S species into soluble higher‐order Li_2_S*_n_*. This is followed by an extended plateau centered around 2.15 V, signifying the continuous and reversible transformation of Li_2_S*_n_* intermediates back to elemental sulfur. The gradual voltage increase near the end of charging indicates the depletion of active species and the completion of sulfur reformation. The discharge profile (Figure 3i) mirrors this multistep mechanism, initiating at approximately 2.6 V and displaying a distinct reduction feature around 2.75 V associated with the conversion of S_8_ to soluble long‐chain Li_2_S*_n_* intermediates. The potential then gradually decreases toward ∼2.1 V, forming a broad plateau corresponding to the further reduction of polysulfides to insoluble Li_2_S_2_/Li_2_S. The smooth and continuous evolution of the potential profile, with minimal polarization between the charge and discharge processes, reflects the efficient redox reversibility of the system. The persistence of these well‐defined plateaus and the absence of voltage distortion over repeated cycles indicate that the TUS‐44@G interlayer effectively regulates polysulfide conversion and diffusion. The TTF moieties facilitate rapid electron exchange, while the conductive graphene framework ensures uniform charge transport and stable interfacial contact. As a result, the TUS‐44@G separator promotes uniform Li_2_S nucleation, suppresses shuttle effects, and enables highly reversible sulfur redox chemistry, thereby contributing to the enhanced coulombic efficiency and cycling stability of the Li–S battery. The long‐term cycling test at a high current density of 5 A g^−1^ (Figure 3j) attests to the durability of the TUS‐44@G interlayer. The cell retains 604 mA h g^−1^ after 1000 cycles from an initial 925 mA h g^−1^, corresponding to a negligible capacity fading of 0.034% per cycle and near‐unity coulombic efficiency. Such exceptional stability and rate resilience position TUS‐44@G as an advanced multifunctional interfacial material capable of simultaneously immobilizing LiPSs, accelerating their redox conversion, and maintaining long‐term electrochemical integrity—paving the way toward practical, high‐performance Li–S batteries.

### 2.4 In Situ Probing of Charge‐Transfer Dynamics and Sulfur Phase Transitions

In situ EIS measurements were conducted to gain deeper insights into the electrochemical dynamics of Li–S batteries and to monitor the evolution of charge‐transfer kinetics and interfacial processes in real time. This technique is particularly powerful for probing the multi‐step solid–liquid–solid conversion of sulfur species, as it sensitively reflects changes in ion transport and interfacial resistance during discharge and charge. As shown in Figure S19, the Nyquist plots reveal distinct impedance evolution during the discharge of the Li–S cell. From 2.7 to 2.3 V, the charge‐transfer resistance gradually increased, consistent with the progressive dissolution of long‐chain LiPSs into the electrolyte. The formation of soluble LiPSs not only enhances electrolyte viscosity but also introduces interfacial heterogeneity, thereby retarding Li^+^ migration. A pronounced impedance jump was observed around 2.3 V, marking the onset of extensive LiPS dissolution and electrolyte saturation. Notably, the TUS‐44@G‐based battery exhibited markedly lower impedance throughout the discharge process compared with the PP separator cell (Figures S13 and S19). This reduction can be attributed to the abundant Lewis‐basic imine nitrogen sites that act as primary Li^+^ anchoring centers, along with catalytically competent redox‐active sites in TUS‐44, which provide strong adsorption anchors for LiPSs, suppressing their uncontrolled shuttling and minimizing viscosity build‐up. As the discharge voltage decreased below 2.1 V, the impedance dropped sharply, corresponding to the precipitation of insoluble Li_2_S. The decline reflects the depletion of soluble LiPSs and the catalytic promotion of their nucleation by TUS‐44, which accelerates LiPS conversion and mitigates kinetic bottlenecks. These observations highlight that while G/PP (Figures S15 and S16) provides moderate suppression of LiPS diffusion, the incorporation of TUS‐44 into the interlayer (Figures S17 and S18) significantly enhances polysulfide confinement and actively facilitates charge‐transfer and redox kinetics across the electrode–electrolyte interface.

To further unravel the structural evolution and redox mechanism of sulfur species, in situ XRD analysis was performed during cycling. This technique enables direct tracking of crystalline phase transformations, offering complementary mechanistic insights into the EIS observations. As depicted in Figure S21, the discharge of the TUS‐44@G‐based battery was accompanied by the gradual disappearance of the characteristic α‐S_8_ diffraction reflections at 23.00°, 25.75°, 26.66°, and 27.64° (JCPDS No. 001‐085‐0799), indicative of the conversion of crystalline sulfur into soluble LiPS intermediates. With further discharge, new reflections corresponding to crystalline Li_2_S emerged, marking the liquid‐to‐solid transition from dissolved polysulfides to the final discharge product. Upon charging, these Li_2_S peaks progressively diminished and ultimately vanished, concomitant with the reappearance of crystalline sulfur peaks, signifying a highly reversible redox transformation. In contrast, the control cell with a PP separator displayed only weak and poorly defined diffraction signatures of both Li_2_S and β‐S_8_, reflecting sluggish kinetics and incomplete sulfur utilization (Figure S22). The stark difference underscores the catalytic role of TUS‐44, which not only stabilizes LiPS intermediates but also lowers the kinetic barrier for both Li_2_S precipitation and oxidation, thereby enabling a more complete and reversible sulfur redox cycle.

### 2.5 DFT‐Informed Understanding of Electronic Structure, Adsorption Energetics, and Redox Pathways

**FIGURE 4:**
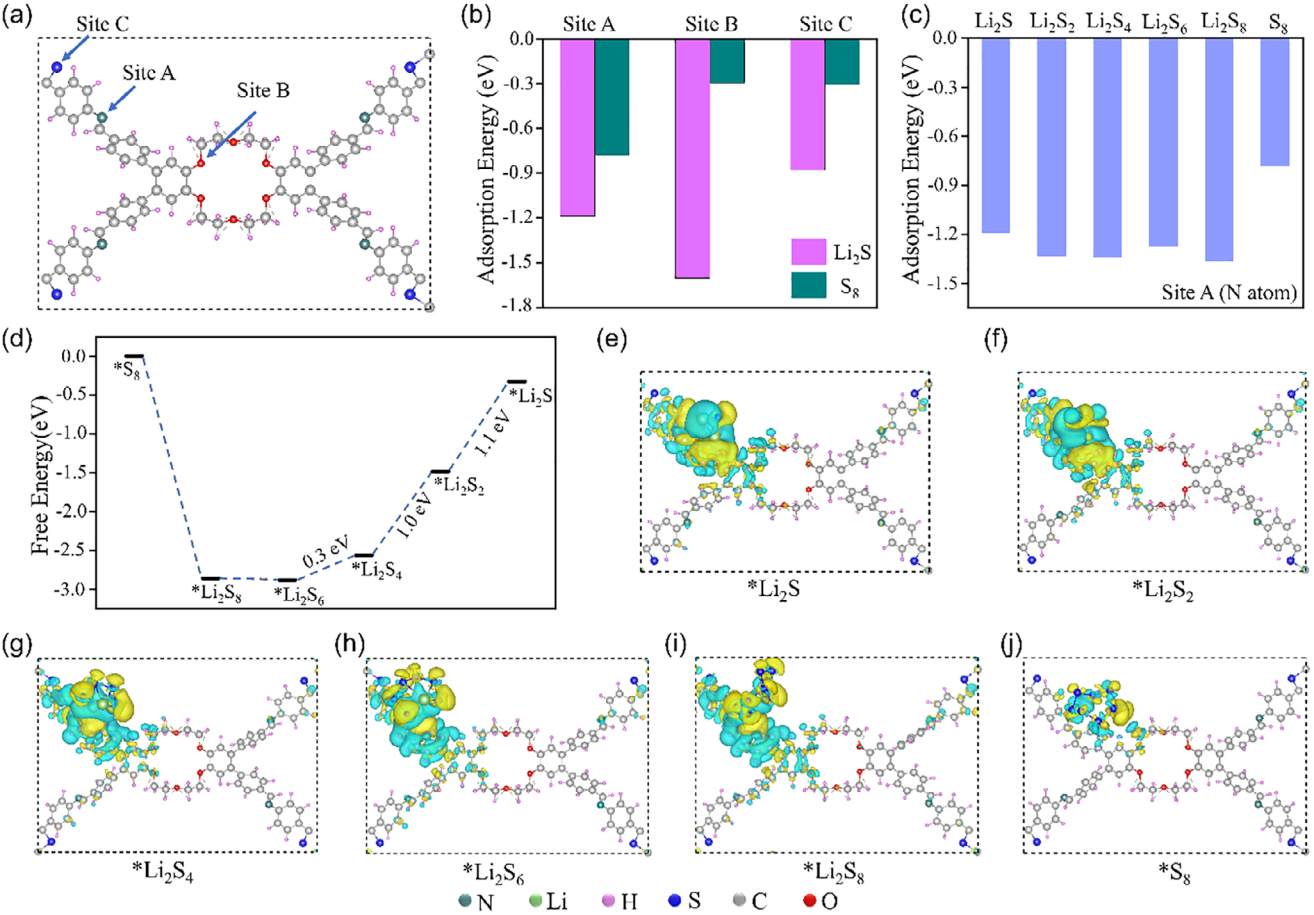
(a) Optimized model structure of TUS‐44. (b) Adsorption energies of Li_2_S and S_8_ for different active sites. (c) Adsorption energies of all Li_2_S*_n_* species for N site. (d) Gibbs free‐energy pathway for the stepwise discharge from S_8_ to Li_2_S. (e–j) charge density plot for all the adsorption.

We performed DFT‐based calculations to study the adsorption energy and electronic structure of sulfur species of the TUS‐44 COF. Initially, the adsorption energy of Li_2_S and S_8_ on the different active sites (Figure 4a), namely site A (N atom), site B (O atom), and site C (S atom), was calculated to identify a favorable active site for Li polysulfide adsorption. Figure 4b shows the corresponding adsorption energies on the considered active sites. The N site exhibits adsorption energies of −1.18 eV for Li_2_S and −0.78 eV for S_8_. In contrast, Li_2_S shows an adsorption energy of −1.80 eV for O atoms and −0.87 eV for S sites; however, for S_8_, the adsorption energies on O (−0.29 eV) and S (−0.30 eV) sites are significantly weaker than those on the N site. Therefore, considering the favorable adsorption for both Li_2_S and S_8_ intermediates, both N and O sites were further studied for subsequent adsorption of lithium polysulfides. Among the two considered active sites, the imine nitrogen atoms within the COF framework exhibit the strongest adsorption affinity toward the lithium polysulfides. The adsorption energy profile for all the polysulfides for the N site and O site is depicted in Figure 4c and Figure S24, respectively. For the O site, short‐chain Li polysulfides (Li_2_S & Li_2_S_2_) show relatively strong adsorption energy, whereas long‐chain Li polysulfides (Li_2_S_6_‐S_8_) exhibit a weak interaction strength. On the other hand, the N site shows moderate and well‐balanced adsorption energies for all the Li polysulfides, ranging from −0.78 to −1.33 eV, which is considered optimal for Li‐S battery performance [66, 67, 68]. To further probe the thermodynamic feasibility of sulfur reduction on TUS‐44, Gibbs free energy profiles were constructed for the stepwise conversion from S_8_ to Li_2_S (Figure 4d) following the sequence [69]: *S_8_+2Li^+^+2e^−^ → *Li_2_S_8_, *Li_2_S_8_ → *Li_2_S_6_ + (1/4)S_8_, *Li_2_S_6_ → *Li_2_S_4_ + (1/4)S_8_, *Li_2_S_4_ → *Li_2_S_2_ + (1/4)S_8_, *Li_2_S_2_ → *Li_2_S + (1/8)S_8_, where * denotes an adsorption site on the COF surface. Taking S_8_ as the reference state (ΔG = 0 eV), the initial reduction step to Li_2_S_8_ is highly exergonic (−2.86 eV), reflecting the thermodynamic driving force for the formation of soluble long‐chain polysulfides. All subsequent steps are also downhill in free energy (Li_2_S_8_ → Li_2_S_6_: −2.88 eV; Li_2_S_6_ → Li_2_S_4_: −2.56 eV; Li_2_S_4_ → Li_2_S_2_: −1.48 eV; Li_2_S_2_ → Li_2_S: −0.32 eV), confirming a favorable cascade toward complete reduction. Importantly, the magnitude of the driving force decreases progressively along the pathway, with the final Li_2_S formation step being the least exergonic. This thermodynamic attenuation is consistent with the experimentally observed sluggish liquid–solid transition and highlights the necessity of catalytic interfaces that can lower kinetic barriers, promote nucleation, and ensure efficient electron transfer during the terminal stages of discharge.

To gain deeper insight into the electronic origins of these interactions, we analyzed the total density of states (TDOS), projected density of states (PDOS), Bader charge distribution, and charge density difference (CDD). The TDOS of pristine TUS‐44 (Figure S25o) reveals a narrow bandgap of ∼0.4 eV, indicative of significantly enhanced electronic delocalization compared to conventional insulating COFs. This reduced bandgap suggests that TUS‐44 possesses intrinsic semiconducting character capable of supporting electron transport during electrochemical cycling. Upon adsorption of Li_2_S*_n_* species, notable modifications in the electronic structure are observed (Figure S25). Specifically, analysis of the p‐orbital contributions shows an increase in p‐state occupancy for heteroatom sites within the framework, accompanied by a systematic shift of the Fermi level toward the conduction band for all adsorption configurations. These changes signify enhanced electronic coupling between the framework and the adsorbates, facilitating charge redistribution across the interface. Such Fermi level modulation is a hallmark of strong adsorbate–substrate interaction and is directly linked to improved charge transfer kinetics during polysulfide redox processes [70, 71]. Bader charge analysis further quantifies the direction and magnitude of charge transfer. As shown, the TTF units consistently act as electron‐accepting centers upon Li_2_S*_n_* adsorption [72, 73]. The extent of charge transfer follows a clear trend, decreasing with increasing polysulfide chain length: 0.50 e (Li_2_S), 0.38 e (Li_2_S_2_), 0.17 e (Li_2_S_4_), 0.15 e (Li_2_S_6_), 0.11 e (Li_2_S_8_), and 0.02 e (S_8_). This trend indicates that short‐chain species exhibit stronger electronic interaction with the framework, which aligns with their higher adsorption energies and preferential stabilization. The stronger charge transfer for Li_2_S and Li_2_S_2_ is particularly important, as it promotes localized electron density accumulation that facilitates nucleation and growth of Li_2_S in a controlled manner rather than random precipitation. The interaction mechanism is further visualized through charge density difference maps (Figure 4e–j). These plots clearly illustrate pronounced electron redistribution upon adsorption, with distinct regions of electron accumulation and depletion at the TTF–polysulfide interface. Electron density accumulation is primarily localized around the heteroatom‐rich framework sites, while depletion is observed in the vicinity of Li atoms within the adsorbates, indicating net charge transfer from Li_2_S*_n_* to the COF. This spatially resolved polarization confirms that the interaction is not purely electrostatic but involves significant electronic coupling and partial covalent character. The consistency between CDD and Bader analyses provides a coherent picture: TUS‐44 acts as an electron‐accepting, redox‐active host governed by a heteroatom interaction hierarchy (N > O > S) that stabilizes polysulfides through coupled charge transfer and orbital interaction. These DFT results establish a unified mechanistic framework in which TUS‐44 simultaneously functions as (i) a thermodynamically optimized adsorption scaffold, (ii) an electronically conductive medium enabling charge transfer, and (iii) a catalytic interface that modulates the energetics of sulfur redox pathways. This synergy underpins the experimentally observed suppression of the shuttle effect, enhanced redox kinetics, and improved cycling stability in Li–S batteries.

### 2.6 Post‐Cycling Lithium Anode Morphology and Pouch‐Cell Demonstration

**FIGURE 5:**
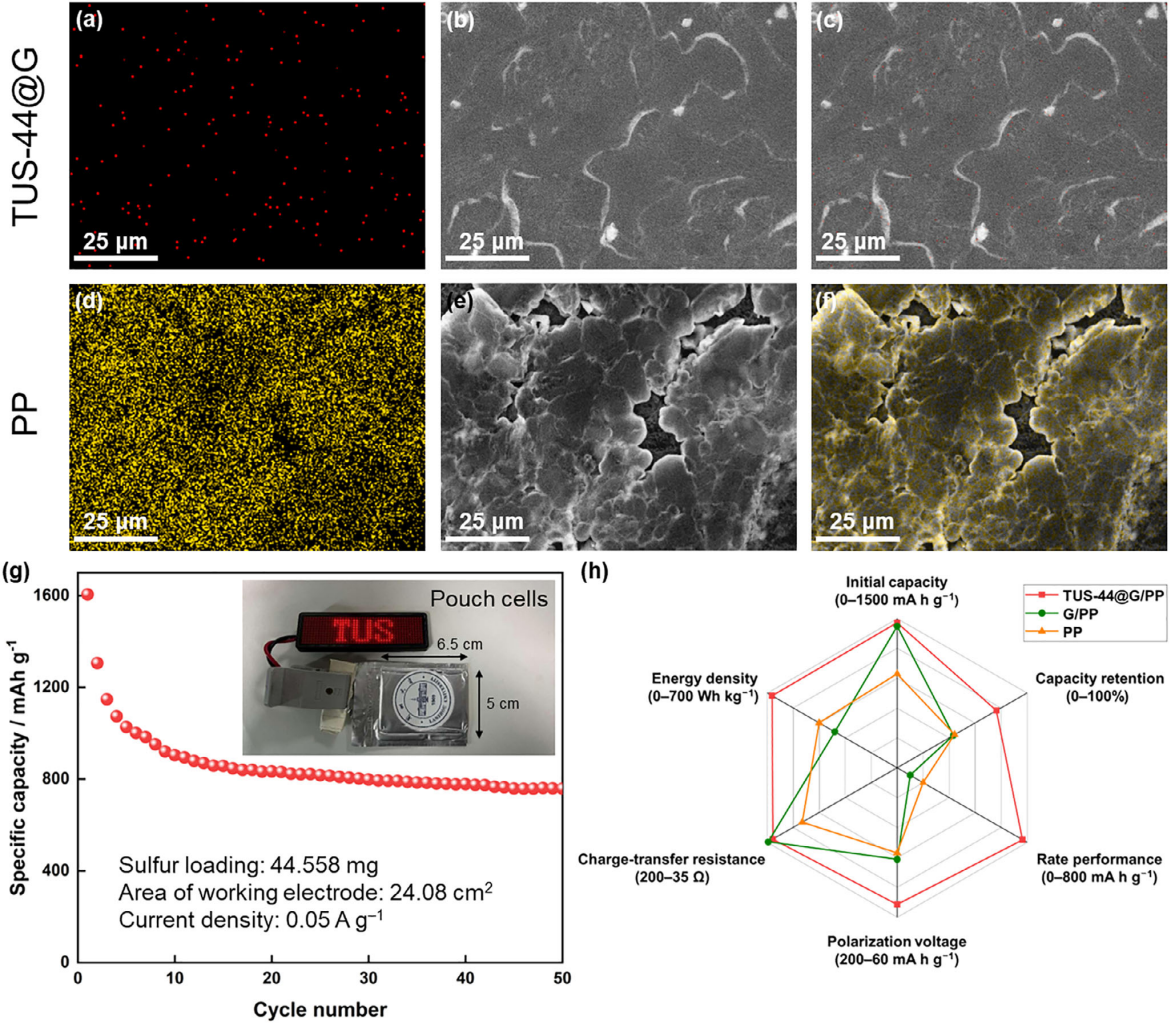
Morphological changes of Li metal anodes and device‐level validation: SEM micrographs, corresponding sulfur EDX maps, and overlays of cycled Li anodes with (b,a,c) TUS‐44@G‐modified vs. (e,d,f) PP separators. (g) Optical photograph (inset) and cycling performance of TUS‐44@G/PP pouch cells. (h) Radar chart illustrating the electrochemical performance of Li–S cells with TUS‐44@G/PP, G/PP, and pristine PP membranes. Parameters include initial capacity, capacity retention, rate capability, polarization voltage, charge‐transfer resistance, and pouch‐cell energy density. The TUS‐44@G/PP system outperforms the controls across all metrics, highlighting its synergistic conversion‐kinetics mediation, rapid Li^+^ transport, and effective suppression of polysulfide shuttling.

To further elucidate the suppression of polysulfide shuttle in working cells, lithium anodes were recovered after 100 cycles and examined by SEM–EDX. In the case of cells with conventional PP separators (Figure 5d,e), the anode surface displayed a rough and heterogeneous morphology with evident dendritic protrusions, which are symptomatic of uneven Li deposition caused by uncontrolled redox processes and shuttle‐induced side reactions. The corresponding EDX analysis revealed strong sulfur signals, confirming substantial crossover and deposition of dissolved polysulfides onto the lithium surface. Such parasitic deposition not only exacerbates the shuttle effect but also accelerates capacity fading and internal resistance growth. In sharp contrast, cells employing the TUS‐44@G interlayer exhibited a markedly smoother and more uniform anode surface after cycling (Figure 5b), with no observable dendritic features. EDX mapping (Figure 5a) further showed only faint sulfur signals, indicating minimal polysulfide deposition. These observations underscore the dual function of the TUS‐44@G layer in both chemically immobilizing LiPS intermediates and catalytically mediating their redox conversion, thereby stabilizing the electrode–electrolyte interface and suppressing deleterious shuttle processes. This morphological protection of the lithium anode directly translates into improved coulombic efficiency, cycling stability, and safety.

The practical relevance of the TUS‐44@G interfacial design was further evaluated through the fabrication of Li–S pouch cells (5.0 × 6.5 cm^2^, effective electrode area: 24.08 cm^2^) incorporating the TUS‐44@G‐modified separator (Figure 5g). The pouch cell was assembled with a sulfur loading of 44.558 mg, corresponding to an areal loading of ∼1.85 mg cm^−2^. The electrolyte volume used for the pouch cell was 0.12 mL, corresponding to an electrolyte‐to‐sulfur (E/S) ratio of 2.69 mL g^−1^. At 0.05 A g^−1^, the cell delivered a high initial gravimetric energy density of ∼674 Wh kg^−1^, calculated using an average discharge voltage of ∼2.1 V. The gravimetric energy density was calculated as *E*  = (*Q*_s_ × *V*_avg_ × *m*_s_) /*m*, where *Q*_s_ is the experimentally measured discharge capacity normalized to sulfur mass, *V*_avg_ is the average discharge voltage, *m*_s_​ is the sulfur mass, and *m*​ is the normalization mass. Based on the electrochemically active stack mass of ∼0.202 g, the calculated energy density is ∼674 Wh kg^−1^. When normalized to the total packaged‐cell mass of ∼0.312 g, including the aluminum‐laminated pouch film (∼0.090 g) and current‐collecting tabs (∼0.020 g), the corresponding packaged‐cell energy density is ∼436 Wh kg^−1^. After 50 cycles, the pouch cell retained an energy density of ∼319 Wh kg^−1^ based on the same active‐stack normalization, demonstrating the structural robustness and sustained electrochemical activity of the TUS‐44@G interface. For comparison, state‐of‐the‐art Li–S pouch cells typically exhibit initial energy densities in the range of ∼300–550 Wh kg^−1^ under practical sulfur loadings and device configurations [74, 75, 76]. Therefore, the performance achieved in this study places the TUS‐44‐based system among the higher‐performing COF‐enabled Li–S pouch‐cell architectures reported to date. Furthermore, the successful operation of the pouch cell in powering LED arrays highlights the scalability and real‐world applicability of this materials platform. Beyond conventional coin‐cell demonstrations, such pouch‐cell validation provides a critical bridge toward practical deployment by linking intrinsic material properties with device‐level performance metrics. A holistic comparison of the TUS‐44@G/PP system with G/PP and pristine PP cells is provided through a radar chart encompassing key performance metrics, including initial discharge capacity, capacity retention, rate capability, polarization voltage, charge‐transfer resistance, and pouch‐cell energy density (Figure 5h). The chart clearly illustrates the superior performance of the TUS‐44@G interface, highlighting its synergistic role in enhancing sulfur utilization, facilitating rapid charge–ion transport, and stabilizing the electrode–electrolyte interface. The high reversible capacity, structural stability of the cycled lithium anodes, and suppression of shuttle collectively establish TUS‐44 as a viable interface material for the industrial deployment of high‐performance Li–S batteries.

## 3 Conclusions

This study establishes a molecular‐level blueprint for engineering polysulfide‐regulating interfaces in Li–S batteries through the deliberate integration of electronic, chemical, and structural functionalities within a single framework. The newly developed COF, TUS‐44, constructed from TTF and benzo[18]crown‐6 linkers, embodies a hierarchically coordinated interaction framework (N > O > S), in which imine nitrogen sites act as primary polysulfide‐binding centers for Li^+^ coordination, crown‐ether oxygen atoms facilitate auxiliary Li^+^ coordination and transport, and sulfur‐rich TTF motifs contribute to electronic coupling and redox mediation, while the π‐conjugated backbone mediates dynamic electron redistribution. When coupled with conductive graphene, the resulting TUS‐44@G hybrid interface synchronizes charge transfer with selective chemisorption, thereby regulating the Li_2_S*_n_* equilibrium and ensuring reversible redox transformations throughout long‐term cycling. The exceptional performance metrics— a high reversible capacity of 1455.7 mA h g^−1^ at 0.2 A g^−1^, superior rate performance of 773 mA h g^−1^ at 10 A g^−1^, and long‐term durability with 0.034% capacity fading per cycle over 1000 cycles at 5 A g^−1—^originate from this synergistic chemical–electronic coupling, which transforms the interfacial region from a passive barrier into an active conversion‐kinetics promoter. Importantly, Li–S pouch cells (5.0 × 6.5 cm^2^, sulfur loading 44.558 mg) incorporating the TUS‐44@G interface attained a remarkable initial energy density of ∼674 Wh kg^−1^ at 0.05 A g^−1^, confirming the design's practical and scalable potential. Beyond Li–S systems, this framework‐directed strategy paves the way for designing next‐generation electrodes and separators that leverage molecular redox centers to steer ion–electron transport at buried interfaces. The insights gained herein open new directions in reticular energy materials by establishing interaction‐hierarchy‐guided design principles for controlling ion–electron coupling at electrochemical interfaces.

## 4 Experimental Section

### 4.1 Synthesis of TUS‐44

B18C6 (23.30 mg, 0.03 mmol) and BDTT (17.06 mg, 0.03 mmol) were ground together and transferred into a Pyrex tube (8 mm i.d., 10 mm o.d.). Anhydrous *o*‐DCB (1.0 mL) was added, and the suspension was sonicated for 10 min. Aqueous acetic acid (6 m, 0.2 mL) was added dropwise, and sonication continued for another 10 min. The tube was cryogenically frozen in liquid nitrogen, degassed by triple freeze–pump–thaw treatment, flame‐sealed under reduced pressure, and maintained at 120°C for 72 h. The solid product was collected by centrifugation, washed thoroughly with THF (5 × 10 mL), subjected to Soxhlet extraction in THF for 24 h, and dried under vacuum at ambient temperature (6 h) and at 100°C (6 h) to give TUS‐44 as a brown powder in 62% yield. Anal. Calcd. for C_20_N_1_H_14_O_2_S_1_: C: 72.28; H: 4.22; N: 4.22; O: 9.64; S: 9.64. Found: C: 72.97; H: 4.33; N: 4.54; O: 7.78; S: 10.39.

### 4.2 Preparation of TUS‐44@G/PP Membranes

TUS‐44@G/PP membranes were produced using a vacuum filtration protocol. In a typical procedure, TUS‐44 (5 mg) and graphene (5 mg) were dispersed in NMP (10 mL) with 1 h of ultrasonication to ensure homogeneity. The suspension was subsequently filtered through a Celgard 2400 (PP) diaphragm, and the resulting composite membrane was dried in vacuo at 60°C overnight to provide a smooth TUS‐44@G/PP membrane with an areal mass loading of 1.4 mg cm^−2^. The membranes were then cut into circular disks of 19 mm diameter. Control samples included G/PP membranes (1.4 mg cm^−2^) prepared analogously and pristine PP membranes. The TUS‐44‐to‐graphene mass ratio of 1:1 was selected to achieve an optimal balance between polysulfide adsorption and electronic conductivity; excessive graphene diminishes chemisorption capability, whereas excessive COF reduces electrical transport. This ratio has previously been shown to deliver superior electrochemical performance for COF@graphene interlayers in Li–S batteries [60].

### 4.3 Preparation of Sulfur Cathodes

The active material (sulfur powder, 70 wt.%), conductive additive (Ketjen black, 20 wt.%), and binder (polyvinylidene fluoride, 10 wt.%) were thoroughly blended, followed by dispersion in NMP to afford a homogeneous slurry. The slurry was coated on aluminum foil with a 150 µm Baker film applicator. Following vacuum drying at 60°C for 24 h, the film thickness was reduced to ca. 100 µm. Circular electrodes (12 mm in diameter) were punched from the films. The aluminum current collector had a thickness of 6 µm, and the areal sulfur loading of the cathodes was approximately 1.5 mg cm^−2^.

### 4.4 Assembly and Electrochemical Analysis of Li–S Cells

Li–S half‐cells (2032 coin type) were assembled in an Ar‐filled glovebox (O_2_ and H_2_O < 0.5 ppm) employing lithium metal as the anode and TUS‐44@G/PP membrane as the separator. The electrolyte consisted of 50 µL of 1.0 m lithium bis(trifluoromethanesulfonyl)imide (LiTFSI) in a 1:1 (v/v) mixture of DOL and DME supplemented with 2 wt.% LiNO_3_ additive. The electrochemical tests were conducted by assembling 2032 coin‐type half‐cells in an argon‐filled glove box with oxygen and water contents below 0.5 ppm. Galvanostatic charge–discharge profiles were recorded on a LAND CT2001A system within a potential window of 1.8–2.7 V at different current densities. CV and EIS experiments were conducted on a BioLogic electrochemical workstation. Specific capacities were normalized to the mass of active sulfur.

### 4.5 DFT Calculations

First‐principles calculations based on density functional theory (DFT) were performed using the Quantum ESPRESSO software package [77, 78, 79]. The exchange–correlation energy was described using the Perdew–Burke–Ernzerhof (PBE) functional within the generalized gradient approximation (GGA) [80, 81, 82]. Electron–ion interactions were treated using scalar‐relativistic ultrasoft pseudopotentials [83]. Brillouin‐zone sampling was carried out with a 3 × 3 × 1 Monkhorst–Pack k‐point grid for both ionic relaxation and electronic self‐consistent field calculations [84]. A plane‐wave cutoff energy of 544 eV (40 Ry) was employed after convergence testing. Structural optimizations were performed using an energy convergence threshold of 1.36 × 10^−3^ eV and a force convergence threshold of 0.026 eV Å^−1^, while the electronic self‐consistency convergence criterion was set to 1.36 × 10^−5^ eV.

The adsorption energy of the complex was determined using the following formula:

$$E\mathrm{ads}\;=E\left(A+B\right)-E\left(A\right)-E\left(B\right)$$

where *E*(A) and *E*(B) denote the energies of the isolated molecules, and *E*(A + B) is the total energy of the complex.

The stepwise lithiation of S_8_ toward Li_2_S was probed through Gibbs free energy profiling by plotting the changes against the reaction coordinate, which displayed the energetic landscape and the thermodynamic feasibility of each transformation.

To derive the DOS and explore the underlying electronic characteristics, the discrete energy levels of this COF system were broadened to curve artificially by employing the Multiwfn wavefunction analysis program [85]. Additionally, the PDOS corresponding to individual elements was computed to enable a more comprehensive interpretation.

## Funding

This study was supported by the Environment Research and Technology Development Fund (JPMEERF20253003) of the Environmental Restoration and Conservation Agency provided by the Ministry of the Environment of Japan, JSPS KAKENHI (grant no. 23H00289 and 23KK0098), the Chubu Electric Power Nuclear Safety Research Institute Research Grant, and the FUSO Innovative Technology Fund.

## Conflicts of Interest

The authors declare no conflicts of interest.

## Supporting information




**Supporting File 1**: smll74240‐sup‐0001‐SuppMat.docx.

**Supporting File 2**: smll74240‐sup‐0002‐cif.zip.

## Data Availability

The data that support the findings of this study are available from the corresponding author upon reasonable request.
