# Supplementary material for: Polysulfide Immobilization and Sulfur Conversion Kinetics Promotion via a Tetrathiafulvalene–Crown Ether COF@Graphene Layer for High‐Rate Lithium–Sulfur Batteries
Source: Small. 2026 Jun 16;22(49):e74240. doi: 10.1002/smll.74240 (PMC13533244; doi:10.1002/smll.74240)
Supplement: Supplementary file 1 [file SMLL-22-e74240-s001.docx]

Supporting Information

Polysulfide Immobilization and Sulfur Conversion Kinetics Promotion via a Tetrathiafulvalene–Crown Ether COF@Graphene Layer for High-Rate Lithium–Sulfur Batteries

Kai Sun,^†,‡^ Tsukasa Irie,^§,‡^ Samim Reza,^#^ Kohki Sasaki,^§^ Mika Nozaki,^§^ Tokuhisa Kawawaki,^§^ Yujun Fu,^†^ Dequan Liu,^†^ Ranjit Thapa,^#,¶,^* Saikat Das,^§,^* Deyan He,^†,^* and Yuichi Negishi^§,^*

^†^School of Materials and Energy, Lanzhou University, Lanzhou, Gansu 730000, China

^§^Institute of Multidisciplinary Research for Advanced Materials, Tohoku University, 2-1-1 Katahira, Aoba-ku, Sendai 980-8577, Japan

^#^Department of Physics, SRM University AP, Amaravati 522 240 Andhra Pradesh, India

^¶^Centre for Computational and Integrative Sciences, SRM University – AP, Amaravati 522 240, Andhra Pradesh, India

^‡^These authors contributed equally to this work

*Corresponding Author

R.T.: [ranjit.t@srmap.edu.in](mailto:ranjit.t@srmap.edu.in)

S.D.: [das.saikat.c4@tohoku.ac.jp](mailto:das.saikat.c4@tohoku.ac.jp)

D.H.: [hedy@lzu.edu.cn](mailto:hedy@lzu.edu.cn)

Y.N.: [yuichi.negishi.a8@tohoku.ac.jp](mailto:yuichi.negishi.a8@tohoku.ac.jp)

**List of Contents**

1. Materials and Methods	S3

2. Structural representation of TUS-44 with bex topology	S5

3. BET surface area plot	S6

4. Scanning electron microscopy (SEM) and transmission electron microscopy (TEM)

characterization	S7

5. Thermogravimetric analysis (TGA)	S10

6. Chemical stability analysis	S11

7. Structural modeling and X-ray diffraction (XRD) analyses	S12

8. LiPSs diffusion experiments	S13

9. Electrolyte wettability	S14

10. In situ electrochemical impedance spectroscopy (EIS)	S15

11. In situ XRD	S18

12. Galvanostatic cycling performance	S20

13. Adsorption energetics and electronic structure evolution upon polysulfide interaction

with TUS-44	S21

14. Crystallographic data	S23

15. Supplementary references	S24

1. Materials and Methods

1.1. Materials. All chemicals and solvents, procured in analytical-grade quality from commercial sources, were utilized as received without further purification unless explicitly noted. Anhydrous *o*-dichlorobenzene, acetic acid and tetrahydrofuran (THF) were purchased from FUJIFILM Wako Pure Chemical Corporation. 4,4',4'',4'''-([2,2'-bi(1,3-dithiolylidene)]-4,4',5,5'-tetrayl)tetraaniline (BDTT) was purchased from ET Co., Ltd. Graphene was purchased from Suzhou Tanfeng Graphene Technology Co., Ltd. *N*-Methyl-2-pyrrolidone (NMP) was purchased from Shanghai Aladdin Biochemical Technology Co., Ltd. Elemental sulfur (S_8_), lithium sulfide (Li_2_S) and lithium nitrate (LiNO_3_) were purchased from Alfa Aesar Chemical Co., Ltd. Ketjen black was purchased from Shenzhen Tianchenghe Technology Co., Ltd. Polyvinylidene fluoride (PVDF) was purchased from Saibo Electrochemical Materials Co., Ltd. Celgard 2400 polypropylene membranes were purchased from Celgard, LLC. Carbon-coated aluminum foil was purchased from Hefei Kejing Material Technology Co., Ltd. 1,3-dioxolane (DOL) was purchased from Macklin Inc. 1,2-dimethoxyethane (DME) and lithium bis(trifluoromethanesulfonyl)imide (LiTFSI) were purchased from Suzhou Duo Duo Chemical Technology Co., Ltd.

1.2. Characterization.

**Powder X-ray diffraction (PXRD).** PXRD measurements were carried out using a Rigaku MiniFlex X-ray diffractometer equipped with a CuKα source (λ = 1.5418 Å) under operating conditions of 40 kV and 15 mA.

Nitrogen sorption. Nitrogen adsorption–desorption isotherms were recorded at 77 K on a Quantachrome Autosorb iQ3 gas sorption analyzer. The COF sample was outgassed at 120 °C for 8 h with a turbomolecular vacuum pump prior to data collection. The Brunauer–Emmett–Teller (BET) method was applied to the adsorption data to calculate the specific surface area, while pore size distributions were derived from the adsorption branch using quenched solid density functional theory (QSDFT, slit/cylindrical pores, N_2_ at 77 K on carbon).

Fourier transform infrared (FT-IR) spectroscopy. FT-IR spectra were acquired with a JASCO FT/IR-6600 FT-IR spectrometer across the 4000–500 cm^−1^ spectral window with samples prepared as KBr pellets.

Solid-state ^13^C cross-polarization magic-angle-spinning (CP-MAS) NMR spectroscopy. Solid-state NMR spectra were collected on a Bruker Biospin Avance III 600 NMR spectrometer equipped with a 4-mm probe, operated at a spinning rate of 10 kHz.

Scanning electron microscopy (SEM). SEM images of the powdered sample and membranes were collected using a JEOL JSM 7000FSHL field emission scanning electron microscope at an accelerating voltage of 5 kV.

High-resolution transmission electron microscopy (HRTEM). HRTEM images with corresponding fast Fourier transform (FFT) and selected area electron diffraction (SAED) patterns were obtained using a JEOL JEM-2100F microscope operated at 80 kV.

Thermogravimetric analysis (TGA). TGA measurements were carried out on a Bruker TG-DTA2010SA instrument with a heating ramp of 10 °C min^-1^ from room temperature to 800 °C under a nitrogen atmosphere (50 mL min^–1^).

Chemical stability test. Solvent stability tests were carried out by dispersing 10 mg of TUS-44 in 8 mL 1,3-dioxolane or 1,2-dimethoxyethane, and storing the suspensions in 10 mL glass vials at room temperature for 7 days. The recovered solids were filtered, washed with THF, and vacuum-dried at 100 °C for 6 h.

X-ray photoelectron spectroscopy (XPS). XPS measurements were performed on a Kratos Axis Ultra DLD spectrometer equipped with a monochromatic Al Kα radiation source (1486.6 eV). All binding energies were calibrated with reference to the C 1s peak at 284.8 eV.

Electrolyte wettability measurements. The wettability of the membrane surfaces toward the liquid electrolyte was evaluated by contact-angle measurements using a SZ-CAMB3 device (Shanghai Xuanjun Instrument Co., Ltd.) equipped with a high-speed CCD camera. A ~5 μL electrolyte droplet was dispensed on the membrane surface, and the static contact angle was determined from images recorded 2 s after droplet deposition using the instrument’s software.

1.3. Synthesis of building blocks. 4,4′,4′′,4′′′-(6,7,9,10,17,18,20,21-octahydrodibenzo[*b*,*k*][1,4,7,10,13,16]hexaoxacyclooctadecine-2,3,13,14-tetrayl) tetrabenzaldehyde (B18C6)^[1,2]^ was synthesized following previous literature.

**2.** **Structural representation of TUS-44 with bex topology**

**Figure S1.** Pore dimensions of TUS-44.

**3.** **BET surface area plot**

**Figure S2**. BET plot for TUS-44 calculated from the N_2_ adsorption isotherms at 77 K. S_BET_ = 516.1 m^2^ g*^−^*^1^, *R*^2^ = 0.999.

**4. Scanning electron microscopy (SEM) and transmission electron microscopy (TEM) characterization**

**Figure S3.** SEM images of TUS-44.

**Figure S4.** TEM image of TUS-44.

**Figure S5.** HRTEM image of TUS-44.

**Figure S6.** SEM images of the (a,b) PP, (c,d) G/PP, and (e,f) TUS-44@G/PP membranes, recorded in both top-view and cross-sectional geometries to reveal morphological distinctions.

**5. Thermogravimetric analysis (TGA)**

**Figure S7.** TGA curve of TUS-44 under N_2_ atmosphere.

**6.** **Chemical stability analysis**

**Figure S8.** PXRD profiles of TUS-44 after immersing in 1,3-dioxolane and 1,2-dimethoxyethane for 7 days.

**7.** **Structural modeling and X-ray diffraction (XRD) analyses**

**Figure S9.** Comparison of experimental XRD pattern (gray) and simulated pattern with AB stacking (blue) for TUS-44.

**Figure S10.** Space-filling model of TUS-44 in the AB stacking mode.

**8.** **LiPSs diffusion experiments**

**Figure S11.** Time-dependent Li_2_S_6_ permeation behavior through the TUS-44@G membrane (top) and pristine PP membrane (bottom).

**9.** **Electrolyte wettability**

**Figure S12.** Electrolyte contact angles of (a) TUS-44, (b) TUS-44@G, and (c) PP membranes. Digital photographs of (d) TUS-44, (e) TUS-44@G, and (f) PP membranes.

**10. In situ electrochemical impedance spectroscopy (EIS)**

**Figure S13.** In situ EIS profiles of PP during discharge process from 2.7 to 1.8 V.

**Figure S14.** In situ EIS profiles of PP during charge process from 1.8 to 2.7 V.

**Figure S15.** In situ EIS profiles of G during discharge process from 2.7 to 1.8 V.

**Figure S16.** In situ EIS profiles of G during charge process from 1.8 to 2.7 V.

**Figure S17.** In situ EIS profiles of TUS-44 during discharge process from 2.7 to 1.8 V.

**Figure S18.** In situ EIS profiles of TUS-44 during charge process from 1.8 to 2.7 V.

**Figure S19.** In situ EIS profiles of TUS-44@G during discharge process from 2.7 to 1.8 V.

**Figure S20.** In situ EIS profiles of TUS-44@G during charge process from 1.8 to 2.7 V.

**11. In situ XRD**

**Figure S21.** In situ XRD profiles of the cells with TUS-44@G-based separator.

**Figure S22.** In situ XRD profiles of the cells with PP separator.

**12.** **Galvanostatic cycling performance**

**Figure S23.** Charge/discharge voltage proﬁles for the 50^th^ cycles at 0.05 A g^−1^ with TUS-44@G separator.

**13. Adsorption energetics and electronic structure evolution upon polysulfide interaction with TUS-44**

**Figure S24.** Adsorption energies of all Li_2_S*_n_* species for O site.

**Figure S25.** Optimized geometries and projected density of states (PDOS) of the N p-orbitals for pristine TUS-44 and polysulfide/S₈-adsorbed TUS-44: (a,b) pristine TUS-44, (c,d) Li_2_S@TUS-44, (e,f) Li_2_S_2_@TUS-44, (g,h) Li_2_S_4_@TUS-44, (i,j) Li_2_S_6_@TUS-44, (k,l) Li_2_S_8_@TUS-44, and (m,n) S_8_@TUS-44. (o) Total density of states (TDOS) of pristine TUS-44.

**14. Crystallographic data**

**Table S1.** Unit cell parameters and fractional atomic coordinates of TUS-44 with **bex** topology in the AA stacking mode.

| Space group: *P*MM2 | | | |
| --- | --- | --- | --- |
| *a* =28.2832 Å, *b* = 19.9426 Å, *c* = 3.6946 Å, *α* = *β* = *γ* = 90.00° | | | |
| C1 | 0.05799 | 0.18236 | -0.83182 |
| C2 | 0.09983 | 0.15151 | -0.75805 |
| C3 | 0.13262 | 0.18107 | -0.55656 |
| C4 | 0.12627 | 0.24323 | -0.42219 |
| C5 | 0.08762 | 0.2784 | -0.51827 |
| C6 | 0.05497 | 0.24878 | -0.73388 |
| N7 | 0.16219 | 0.27038 | -0.22075 |
| C8 | 0.3104 | 0.46451 | 0.51388 |
| C9 | 0.27296 | 0.42114 | 0.39158 |
| C10 | 0.23049 | 0.43184 | 0.52151 |
| C11 | 0.19382 | 0.40327 | 0.36716 |
| C12 | 0.19953 | 0.34895 | 0.14612 |
| C13 | 0.24264 | 0.32466 | 0.08151 |
| C14 | 0.27937 | 0.36179 | 0.19713 |
| C15 | 0.16022 | 0.32358 | -0.0332 |
| C16 | 0.02283 | 0.14322 | -1.0359 |
| S17 | 0.95231 | 0.07265 | -1.25482 |
| C18 | 0.3484 | 0.56804 | 0.6366 |
| C19 | 0.38608 | 0.53434 | 0.7589 |
| O20 | 0.42404 | 0.56709 | 0.88936 |
| C21 | 0.42032 | 0.63546 | 0.98153 |
| C22 | 0.46226 | 0.65671 | 1.17305 |
| H23 | 0.10804 | 0.10169 | -0.83124 |
| H24 | 0.16305 | 0.15461 | -0.49823 |
| H25 | 0.08386 | 0.32989 | -0.45302 |
| H26 | 0.03232 | 0.28105 | -0.86211 |
| H27 | 0.22609 | 0.45303 | 0.78114 |
| H28 | 0.16101 | 0.41972 | 0.44776 |
| H29 | 0.24792 | 0.28099 | -0.08174 |
| H30 | 0.31211 | 0.34416 | 0.13122 |
| H31 | 0.12976 | 0.35148 | -0.01348 |
| H32 | 0.34808 | 0.62086 | 0.64062 |
| H33 | 0.39219 | 0.64319 | 1.16857 |
| H34 | 0.41389 | 0.66754 | 0.74563 |
| H35 | 0.46514 | 0.62036 | 1.3946 |
| H36 | 0.45824 | 0.70767 | 1.27908 |
| C37 | 0 | 0.03747 | -1.46029 |
| O38 | 0.5 | 0.65288 | 0.94696 |

**15. Supplementary references**

[1] X. Gu, B. Wang, Y. Pang, H. Zhu, R. Wang, T. Chen, Y. Li, X. Yan, *New J. Chem.* **2023**, *47*, 2040.
[2] D.-W. Lim, S. A. Chyun, M. P. Suh, *Angew. Chem., Int. Ed*. **2014**, *53*, 7819.
